# Prevalence and Risk Factors of Retinopathy of Prematurity Among the Infants Screened at a Tertiary Level Hospital in Central India: A Cross-Sectional Study

**DOI:** 10.7759/cureus.114318

**Published:** 2026-08-10

**Authors:** Kanika Singh, Chetan Khare, Priti Singh, Samendra Karkhur, Vidhya Verma, Saroj Gupta

**Affiliations:** 1 Ophthalmology, All India Institute of Medical Sciences Bhopal, Bhopal, IND; 2 Neonatology, All India Institute of Medical Sciences Bhopal, Bhopal, IND

**Keywords:** aggressive retinopathy of prematurity, awareness of rop, central india region, laser in rop, low birth-weight, prematurity complications, prevalence of rop, retinopathy of prematurity, retinopathy of prematurity (rop), risk factors for retinopathy of prematurity

## Abstract

Purpose: To determine the prevalence and risk factors for retinopathy of prematurity (ROP) in infants screened at a tertiary level hospital in central India, and to assess the level of ROP awareness among the parents/guardians with the help of a self-designed questionnaire.

Methods: A cross-sectional study over 18 months included 252 infants screened for ROP. Risk factors were recorded, and parents/guardians completed a questionnaire. Statistical analysis done using Statistical Package for the Social Sciences (SPSS) version 22.

Results: Among the 252 infants screened, 86 (34.1%) were diagnosed with ROP. Of the total cohort, nine (3.6%) had type 1 ROP, 14 (5.6%) had aggressive retinopathy of prematurity (AROP), seven (2.8%) had hybrid ROP, and 10 (4.0%) had advanced stage 4 or 5 disease. The mean gestational age (GA) and birth weight in ROP infants was observed to be 30.59 ± 3.12 weeks and 1215.01 ± 309.54 grams, respectively. Low birth weight (LBW), prematurity, respiratory distress syndrome (RDS), neonatal intensive care unit (NICU) stay >2 weeks, oxygen supplementation >1 week and phototherapy are the significant risk factors for ROP, observed in this study. Out of 252, only 30 guardians/parents had heard of ROP and were aware of its blinding nature (11.9%), 28 knew that prematurity and LBW are the potential risk factors for development of ROP (11.1%), and 27 were aware that it can be treated (10.7%).

Conclusion: Due to highly variable neonatal care, regional ROP screening guidelines should be followed for timely diagnosis and management of ROP to achieve optimal outcome. There is a need for specific programs for ROP awareness among parents and healthcare staff to emphasize the need for timely screening, appropriate scheduling, consistent follow-up and the availability and effectiveness of various treatment modalities, in order to prevent this potentially avoidable cause of blindness.

## Introduction

Retinopathy of prematurity (ROP) is a proliferative retinal vascular disease of premature, low birth weight (LBW) infants. Early exposure to high ambient oxygen concentration is considered a key factor in the pathogenesis of ROP that initially causes retardation in vessel growth due to hyperoxia and subsequent hypoxia leads to release of vascular endothelial growth factor and other angiogenic molecules resulting in anomalous neovascularization of retina leading to various stages of ROP. The disease spectrum ranges from spontaneous resolution to severe complications such as retinal detachment and bilateral blindness. ROP is one of the world’s leading causes of preventable blindness in infants. Effective management requires timely screening, accurate diagnosis, and prompt treatment through coordinated efforts between neonatologists and ophthalmologists. India is currently experiencing the so-called "third epidemic" of ROP, driven by several contributing factors such as increased survival of preterm infants, suboptimal neonatal intensive care unit (NICU) practices, and limited access to ROP screening and treatment services. The first epidemic occurred due to the unregulated use of supplemental oxygen, while the second epidemic was observed in high-income countries with improved survival of very preterm neonates [1].

The epidemiology of ROP varies geographically, largely influenced by regional disparities in the quality and standards of neonatal care practices. These differences affect not only the incidence but also the severity and outcomes of ROP across various settings [2]. There is limited region-specific data from the central region of India regarding the prevalence, risk factors, and parental awareness of ROP. Most available evidence is derived from other regions of the country, which may not fully reflect the demographic profile, neonatal care practices, and healthcare access in this region. Additionally, there is a lack of objective assessment on level of awareness among the parents regarding ROP in India.

ROP is classified according to the International Classification of Retinopathy of Prematurity (ICROP), Third Edition, based on the retinal zone involved, disease stage, extent in clock hours, the presence or absence of plus disease, and aggressive retinopathy of prematurity (AROP). This standardized classification guides disease severity assessment, treatment decisions, and follow-up [3].

This study aims to determine the prevalence of ROP and to identify the associated risk factors for the development of ROP among the infants screened at a tertiary level hospital in central India (inborn and outborn referrals). This also provides valuable insights into the quality of neonatal care provided in this region. Furthermore, it seeks to assess the level of awareness among parents and caregivers regarding ROP, its risk factors, and the importance of timely follow-up using a self-designed questionnaire. The findings may help identify gaps in awareness and inform future educational and preventive strategies aimed at improving ROP-related outcomes.

## Materials and methods

This 18-month cross-sectional study was done at the Department of Ophthalmology, All India Institute of Medical Sciences Bhopal, from September 2023 to February 2025. Both inborn and outborn infants referred to our tertiary center for ROP screening were enrolled in the study at their first visit. All infants were screened according to the Rashtriya Bal Suraksha Karyakram (RBSK) 2017 guidelines, including: birth weight <2,000 g, or gestational age (GA) <34 weeks, or GA between 34 and 36 weeks but with systemic comorbidities such as cardiorespiratory support, prolonged oxygen therapy, respiratory distress syndrome (RDS), chronic lung disease, fetal hemorrhage, blood transfusion, neonatal sepsis, exchange transfusion, intraventricular hemorrhage (IVH), apneas, and poor postnatal weight gain or, infants with an unstable clinical course who are at high risk (as determined by the neonatologist or pediatrician) and diagnosed according to the latest ICROP, Third Edition [3].

Screening was performed in outpatient departments (OPDs) and NICU under proper aseptic conditions under pediatrician guidance. Standard indirect ophthalmoscopic examination following pharmacological pupillary dilatation was performed. Infants were monitored during and after the examination for any procedure-related adverse events. No screening-related complications or adverse events were observed during the study period.

Diagnosis was recorded as ‘No ROP’, ‘type 1 ROP’, ‘type 2 ROP’, ‘AROP’ or ‘advanced ROP’. Detailed history and clinical course of the infants noted and parents assessed for the awareness using the self-designed questionnaire including following questions (in both English and Hindi) (Table 1). 

**Table 1:** Self-designed questionnaire to assess parents for awareness of retinopathy ROP: Retinopathy of prematurity; LBW: Low birth weight

S. No.	Question	Response
1	Have you heard of ROP before coming to AIIMS Bhopal?	Yes/No
2	From where did you get to know about ROP?	
3	Do you know LBW and prematurity predispose to ROP?	Yes/No/Can’t say
4	Do you know ROP can cause blindness?	Yes/No/Can’t say
5	Do you know this disease is treatable?	Yes/No/Can’t say

One questionnaire was completed for each enrolled infant. In cases of multiple births (twins or triplets), the parent/guardian completed a separate questionnaire for each infant resulting in 252 questionnaire responses corresponding to the 252 infants included in the study.

After completion of the questionnaire and ophthalmic examination of the infant, all parents/guardians were counseled regarding ROP, the importance of timely screening, treatment, and adherence to follow-up. This educational intervention was provided after the baseline assessment of awareness was done.

Statistical analysis

Statistical analysis was carried out using statistical packages for IBM Statistical Package for the Social Sciences (SPSS) vs 22 for Windows. Continuous and categorical variables are expressed as mean ± SD and percentages, respectively. Descriptive statistics is done for prevalence of ROP, gender, outborn/inborn, NICU/OPD, singlet/twin/triplet. Chi-square test is used to compare outborn/inborn among gender, outborn/inborn among NICU/OPD, outborn/inborn among types of ROP. Binomial logistic regression is done to assess risk factors associated with ROP. Two-sided p values are considered as statistically significant at p<0.05.

## Results

This study conducted at AIIMS Bhopal from September 2023 to February 2025 included 252 infants out of which 71 were screened in NICU and 181 came to OPD for screening. Out of 252 children, 57.1% (n=144) were male and 42.9% (n=108) were female. The gender distribution in ROP infants was observed to be 51.1% (n=45) male and 48.9% (n=43) female. Out of 252 children, four were triplets, 53 were twins and the rest were single born.

**Figure 1:**
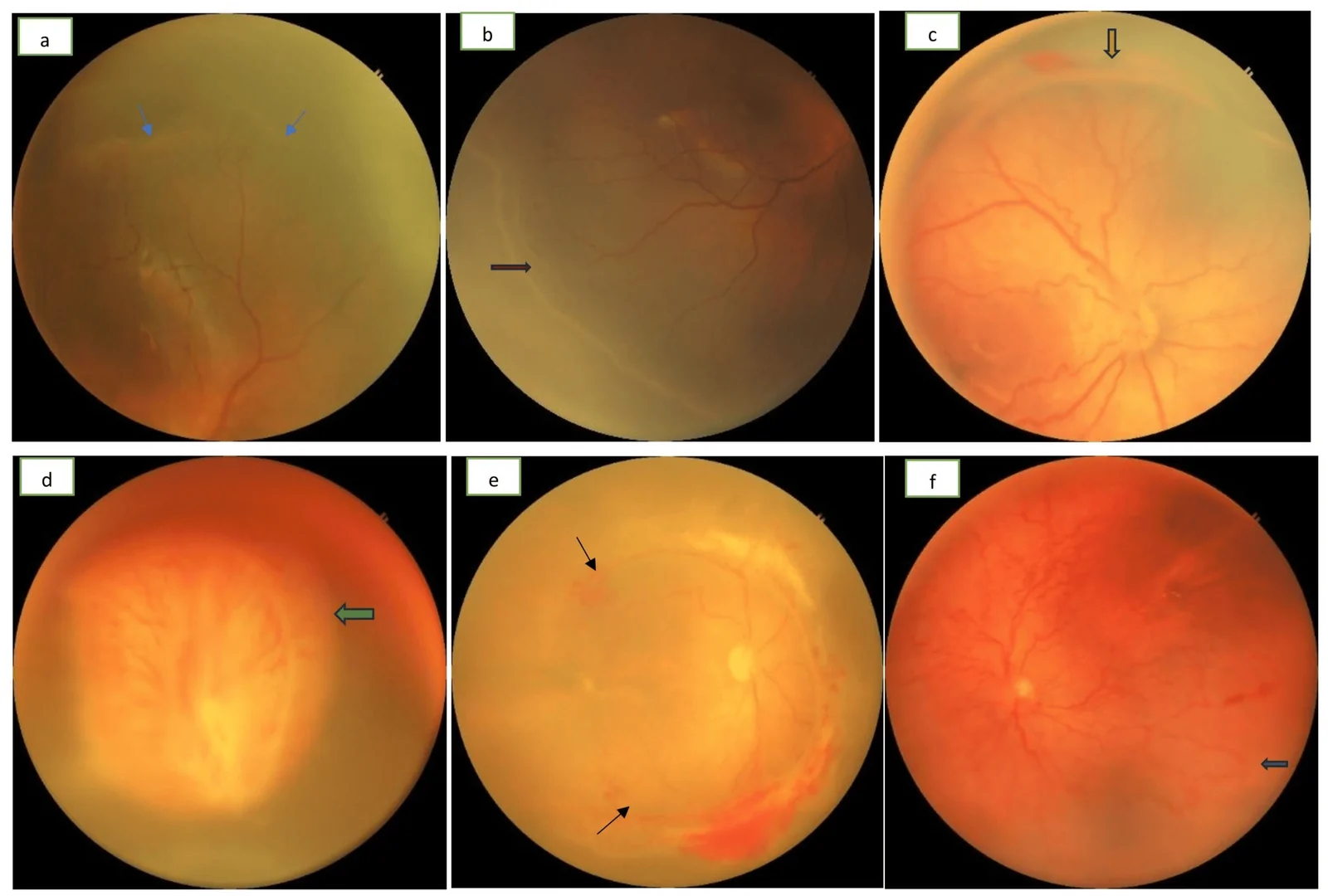
Spectrum of ROP observed Image courtesy: ROP Clinic, Department of Ophthalmology, AIIMS, Bhopal a) Stage 1 ROP (blue arrows); b) Stage 2 ROP showing ridge (purple solid arrow); c) Stage 3 ROP showing extraretinal fibrovascular membrane (yellow solid arrow); d) Stage 5 ROP showing complete retinal detachment (green solid arrow); e) Hybrid ROP showing staged disease and AROP (black arrows); f) AROP showing vascular loops and flat neovascularization (blue solid arrow) ROP: Retinopathy of prematurity; AROP: Aggressive retinopathy of prematurity

Out of 252 infants, 86 had ROP (prevalence= 34.1%). Figure 1 represents the spectrum of ROP disease observed in this study.

Among the infants diagnosed with ROP, the disease distribution was as follows: 46 (18.3%) had type 2 ROP, nine (3.6%) had type 1 ROP, 14 (5.6%) had AROP, seven (2.8%) had hybrid ROP, and 10 (4.0%) had advanced ROP (Stages 4 and 5) (Table 2). 

**Table 2:** Prevalence of different types of ROP ROP: Retinopathy of prematurity; AROP: Aggressive retinopathy of prematurity

Type of ROP	N=252	%
No ROP	166	65.9
Type 1 ROP	9	3.6
Type 2 ROP	46	18.3
AROP	14	5.6
Hybrid ROP	7	2.8
Advanced ROP	10	4.0

The overall mean GA of all the infants screened for ROP was 32.71 ± 3.76 weeks whereas that for ROP infants was found to be 30.59 ± 3.12 weeks (Table 3). The overall mean birth weight of all screened infants is found to be 1501.98 ± 513.37 grams and the mean birth weight of ROP infants is 1215.01 ± 309.54 grams (Table 4).

**Table 3:** Mean GA in types of ROP ROP: Retinopathy of prematurity; AROP: Aggressive retinopathy of prematurity; GA: Gestational age

Type of ROP	Mean GA (weeks)	SD
No ROP	33.77	3.63
ROP	30.59	3.12
Type 1 ROP	30.00	2.00
Type 2 ROP	30.69	2.84
AROP	31.21	4.40
Hybrid ROP	30.71	3.86
Advanced ROP	30.40	2.91
Overall Mean GA	32.71	3.76

**Table 4:** Mean birth weight in types of ROP ROP: Retinopathy of prematurity; AROP: Aggressive retinopathy of prematurity

Type of ROP	Mean Birth Weight (grams)	SD
No ROP	1,644.88	538.72
ROP	1,215.01	309.54
Type 1 ROP	1,243.33	222.82
Type 2 ROP	1,227.32	358.32
AROP	1,181.84	242.64
Hybrid ROP	1,199.28	298.19
Advanced ROP	1,254.00	243.91
Overall birth weight	1,501.98	513.37

In the NICU, 81.7% infants (n=58) were inborn, which is a significant number, while 18.3% (n=13) were outborn; in the OPD, 56.9% (n=103) were inborn, while 43.1% (n=78) were outborn. Among the inborn infants, a majority (85.7% (138 infants)) did not exhibit any signs of ROP. Type 2 ROP was observed in 13.7% (22 infants), while only one infant was diagnosed with type 1 ROP (0.6%) requiring urgent treatment. In contrast, all severe forms of ROP were found exclusively in outborn infants, specifically, 11% (10 infants) presented with advanced stage (Stage 4 or 5) ROP, 7.7% (seven infants) had hybrid form of ROP, 15.4% (14 infants) had AROP, 8.8% (eight infants) had type 1 ROP requiring intervention and only 26.4% (24 infants) and 30.8% (28 infants) had type 2 and no ROP, respectively (Table 5).

**Table 5:** Distribution of types of ROP among inborn/outborn infants ROP: Retinopathy of prematurity; AROP: Aggressive retinopathy of prematurity

Type of ROP	Inborn		Outborn		Total	
	N=161	%	N=91	%	N=252	%
No ROP	138	85.7	28	30.8	166	65.9
Type 1 ROP	1	0.6	8	8.8	9	3.6
Type 2 ROP	22	13.7	24	26.4	46	18.3
AROP	0	0.0	14	15.4	14	5.6
Hybrid ROP	0	0.0	7	7.7	7	2.8
Advanced ROP	0	0.0	10	11.0	10	4.0

Among the various risk factors evaluated, LBW, prematurity, RDS, and NICU stay more than two weeks were found significant (p<0.05) in ROP group, whereas oxygen administration for more than one week and phototherapy were found highly significant (p=0.001). Table 6 demonstrates all the risk factors for ROP evaluated in this study. Table 7 demonstrates the individual chi square, degree of freedom, odds ratio for each risk factor considered in this study.

**Table 6:** Risk factors for ROP * implies p value< 0.05 and significant values; ** implies p value<0.001 and highly significant values ROP: Retinopathy of prematurity; CI: Confidence interval; LBW: Low birth weight; SGA: Small for gestational age; RDS: Respiratory distress syndrome; NICU: Neonatal intensive care unit; NEC: Necrotizing enterocolitis; IVH: Intraventricular hemorrhage; PDA: Patent ductus arteriosus

Risk Factor	Presence	ROP		No ROP		Total	Odds Ratio	95% CI		p-value
		N=88	%	N=164	%			Lower	Upper	
LBW	Absent	1	1.1	14	8.5	15	12.29	1.345	112.3	0.026*
	Present	87	98.9	150	91.5	237				
Prematurity	Absent	1	1.1	29	17.7	30	9.38	1.145	76.876	0.037*
	Present	87	98.9	135	82.3	222				
SGA	Absent	27	45.8	76	59.4	103	1.73	.930	3.226	0.082
	Present	32	54.2	52	40.6	84				
RDS	Absent	39	44.8	107	65.2	146	2.33	1.063	5.134	0.035*
	Present	48	55.2	57	34.8	105				
Asphyxia	Absent	85	96.6	161	98.2	246	0.69	.066	7.226	0.759
	Present	3	3.4	3	1.8	6				
NICU Stay	<2 weeks	21	23.9	85	51.8	106	2.30	1.230	4.319	0.009*
	>2 weeks	67	76.1	79	48.2	146				
O_2_ Therapy	<1 week	54	62.1	152	92.7	206	7.56	3.336	17.145	<0.001**
	>1 week	33	37.9	12	7.3	45				
Shock	Absent	87	98.9	164	100	251		0.00	-	1.00
	Present	1	1.1	0	0.0	1				
NEC	Absent	87	98.9	163	99.4	250	0.28	.005	15.825	0.543
	Present	1	1.1	1	0.6	2				
IVH	Absent	87	98.9	161	98.2	248	0.59	.047	7.478	0.688
	Present	1	1.1	3	1.8	4				
PDA	Absent	85	96.6	160	97.6	245	1.99	.275	14.391	0.495
	Present	3	3.4	4	2.4	7				
Hypoglycemia	Absent	88	100.0	159	97.0	247	0.00	.000	--	1.00
	Present	0	0.0	5	3.0	5				
Septicemia	Absent	80	90.9	147	89.6	227	1.04	.282	3.880	0.947
	Present	8	9.1	17	10.4	25				
Hyperbilirubinemia	Absent	77	87.5	152	92.7	229	1.81	.764	4.288	0.158
	Present	11	12.5	12	7.3	23				
Blood Transfusion	Absent	85	96.6	164	100	249		0.00	-	1.00
	Present	3	3.4	0	0.0	3				
Phototherapy	Absent	76	86.4	161	98.2	237	8.47	2.32	30.913	0.001*
	Present	12	13.6	3	1.8	15				
Anemia	Absent	86	97.7	158	96.3	244	0.26	0.03	2.319	0.232
	Present	2	2.3	6	3.7	8				

**Table 7:** Risk factors with df and odds ratio Interpretation: 0.1=Small, 0.3=Moderate, 0.5=Large *p < 0.05: significant; **p < 0.001: highly significant Odds ratios with 95% CI are from binomial logistic regression df: Degree of freedom; CI: Confidence interval; LBW: Low birth weight; SGA: Small for gestational age; RDS: Respiratory distress syndrome; NICU: Neonatal intensive care unit; NEC: Necrotizing enterocolitis; IVH: Intraventricular hemorrhage; PDA: Patent ductus arteriosus

Risk Factor	χ² (df)	p- value	Effective Size (Φ)	Odds Ratio (95% CI)
LBW	5.60 (1)	0.018*	0.15 (small–mod)	12.29 (1.35 – 112.3)
Prematurity	14.95 (1)	<0.001**	0.24 (moderate)	9.38 (1.15 – 76.9)
SGA	3.02 (1)	0.082	0.11 (small, ns)	1.73 (0.93 – 3.23)
RDS	9.74 (1)	0.002*	0.20 (moderate)	2.33 (1.06 – 5.13)
Asphyxia	0.61 (1)	0.433	0.05 (none)	0.69 (0.07 – 7.23)
NICU stay >2 weeks	18.38 (1)	<0.001**	0.27 (mod–large)	2.30 (1.23 – 4.32)
O₂ therapy >1 week	36.21 (1)	<0.001**	0.38 (large)	7.56 (3.34 – 17.1)
Shock	1.87 (1)	0.171	0.09 (none)	0.00 (–)
NEC	0.20 (1)	0.653	0.03 (none)	0.28 (0.01 – 15.8)
IVH	0.18 (1)	0.675	0.03 (none)	0.59 (0.05 – 7.48)
PDA	0.20 (1)	0.655	0.03 (none)	1.99 (0.28 – 14.4)
Hypoglycemia	2.74 (1)	0.098	0.10 (small, ns)	1.04 (0.28 – 3.88)
Septicemia	0.10 (1)	0.747	0.02 (none)	–
Hyperbilirubinemia	1.85 (1)	0.173	0.09 (none)	1.81 (0.76 – 4.29)
Blood Transfusion	5.66 (1)	0.017*	0.15 (small–mod)	–
Phototherapy	14.26 (1)	<0.001**	0.24 (moderate)	8.47 (2.32 – 30.9)
Anemia	0.36 (1)	0.550	0.04 (none)	0.26 (0.03 – 2.32)

Out of the total 252 participants, only 30 (11.9%) had prior awareness of ROP, primarily through hospital staff during previous NICU stay or in cases where the parents were themselves involved in medical field. In one instance, knowledge was obtained through independent Internet search. 30 were aware about the blinding potential of ROP, 28 were aware that LBW and prematurity predispose infants to ROP, and 27 were aware that ROP can be treated if diagnosed in a timely manner (Table 8).

**Table 8:** Response of the questionnaire on awareness ROP: Retinopathy of prematurity; LBW: Low birth weight

No.	Question	Answer	N=252	%
Q1	Parents have heard of ROP	Yes	30	11.9
		No	222	88.1
Q2	From where	NA	222	88.1
		Doctor	3	1.2
		Google search	1	0.4
		Hospital	21	8.3
		Nursing	3	1.2
		Relatives	2	0.8
Q3	Do you know prematurity and LBW predispose to ROP?	Can’t say	11	4.4
		No	213	84.5
		Yes	28	11.1
Q4	Do you know it causes blindness?	Can’t say	7	2.8
		No	215	85.3
		Yes	30	11.9
Q5	Do you know it is treatable?	Can’t say	16	6.3
		No	209	82.9
		Yes	27	10.7

## Discussion

This cross-sectional study, conducted at the Department of Ophthalmology, AIIMS Bhopal, recruited 252 participants undergoing ROP screening to determine the prevalence of ROP in this part of the country, to identify the risk factors of ROP observed here and to determine the awareness of ROP in the parents/guardians with the help of a self-designed questionnaire.

Out of all 252 infants, 57.1% (n=144) were male and 42.9% (n=108) were female. However, no gender predilection was observed in the prevalence of ROP (51.1% (n=45) ROP in male infants and 48.9% (n=43) in female infants).

Overall, postmenstrual age and weight of all infants at presentation was 37.97 ± 8.68 weeks and 1,983.28 ± 835.10 grams, respectively. The overall mean GA of the infants in the study was 32.71 ± 3.76 weeks. Among infants diagnosed with ROP, the mean GA was lower, at 30.59 ± 3.12 weeks. Similarly, the mean birth weight of all infants was 1,501.98 ± 513.37 grams, whereas infants with ROP had a lower mean birth weight of 1,215.01 ± 309.54 grams.

The overall prevalence of ROP in the study population was 34.1% (86 out of 252 infants). Among these, the prevalence of type 1 ROP was 3.6%, AROP was 5.6%, hybrid ROP was 2.8% and advanced stage ROP (Stage 4 or 5) was observed in 4.0% of cases. All the severe forms of ROP were found exclusively in outborn infants referred from other NICUs, while only one type 1 ROP (0.6%) requiring urgent intervention was observed among the inborn infants screened. 85.7% (138 out of 161 inborn infants) of them did not develop any ROP. 

Similar studies conducted in western India reported the prevalence of ROP to be 26.9% (45 out of 167 infants) [4] and 19.28% (54 out of 280 babies) [5], which are lower as compared to the prevalence observed in our study from central India, i.e., 34.1% (86 out of 252 infants) .

A study from eastern region of Madhya Pradesh reported the prevalence of ROP to be 30% [6], comparable to our study. The prevalence of APROP in their study was 27.7% [6], which is higher than that in our study (i.e. 5.6%). Additionally, 16.6% had advance ROP [6], also higher than that observed in our study (i.e., 4.0%). This indicates the highly variable neonatal care given in different parts of the same state. The mean GA and birth weight (30.9 weeks and 1,359.9 gm, respectively) of AROP patients is also different from our study (31.21 weeks and 1181.84 gm respectively). Another study conducted in Madhya Pradesh reported an incidence of ROP at 23.4%, which is lower than the prevalence observed in our study [7].

In a study conducted in north India, the prevalence of threshold or worse ROP was reported to be 45.1% [8], which is higher than the prevalence observed in our study (15.87%).

In a study conducted in Kerala, including infants with GA ≤28 weeks or birth weight ≤ 1,000 gm, the prevalence of any ROP was reported to be 87% [9], significantly higher than the prevalence observed in our study. This is probably because in this study, only the most susceptible infants were considered. 19.14% of these infants required intervention [9]. Another study in Kerala reported the prevalence of ROP to be 25.36% [10], which is lower than that in our study (34.1%). Out of these ROP infants, 9.95% had type 1 disease, and out of these type 1 disease infants, 20.73% eyes had AROP. In contrast, in our study, type 1 disease was observed in 3.6% infants and AROP in 5.6% infants. This points out that the overall prevalence is more in our study but the prevalence of treatment requiring disease is less. However, in this study, no infant came with advanced stage disease, unlike ours where 4% infants presented with Stage 4 or 5 disease. Another study from Karnataka reported the incidence of any ROP to be 21.6%, while severe ROP was observed in 6.7% of the infants [11], which is lower than that observed in our study.

In a study conducted to review the incidence and related risk factors of ROP across various countries, it was found that worldwide prevalence of ROP is difficult to estimate due to a lack of any standardized ROP screening protocol and registry of data worldwide [12]. The study also found that only a few countries, including Kenya, Nigeria, India, the United States, and Romania, have a screening protocol for ROP. This study observed that in most of the worldwide studies on ROP, it is most prevalent in preterm or LBW infants. They also found that the reason for increasing mortality associated with this condition is a lack of screening protocols and utilities in certain countries. They suggested that associated morbidity and mortality can be reduced by implementing proper screening and strict treatment protocols with controlled oxygen usage and availability of resources in hospitals to adequately diagnose and manage ROP as soon as possible. Another similar study on global prevalence and severity trends of ROP involving 121,618 premature infants over four decades reported the pooled prevalence of ROP to be 31.9% [13], consistent with our study. Also, severe ROP was observed in 7.5% of infants [13]. However, no significant difference was observed in ROP prevalence over these four decades (between January 1985 and December 2021). The study observed the highest prevalence of ROP in premature infants with ≤ 28 weeks of GA [13]. It also noted that the highest ROP prevalence and mortality rates are present in lower-middle income countries [13]. In contrast, the highest severe ROP prevalence was found in high-income countries [13].

Although these studies generally adhered to the prevailing national ROP screening guidelines during their respective study periods and used the ICROP for disease classification, with minor institutional variations where applicable, differences in study design (most being retrospective observational studies, whereas ours is cross-sectional), patient recruitment, and neonatal care practices should be considered when interpreting these comparisons.

A study from Portugal neonatal units involving 475 infants of median GA of 30 weeks and median birth weight of 1,229 grams, similar to our study, reported ROP in 23.8% infants [14], lesser than the prevalence observed in our study, and 6.1% of these were treated.

In a study from Turkey, any stage of ROP among 2,186 infants was reported to be 43.5%; these rates were 81.1% in extremely LBW infants (≤1,000 gm) and 92.9% in extremely low GA (≤25 weeks) [15].

A study from Brazil involving 602 neonates with a GA ≤32 weeks or birth weight ≤1,500 grams, or GA between 32 and 37 weeks or a birth weight >1,500 grams but with associated comorbidities (including multiple gestation, RDS, sepsis, blood transfusions, or IVH reported an incidence of any ROP of 33.9% [16], which was comparable to that observed in our study (i.e., 3.6%). Also, type 1 prethreshold ROP was observed in 5.0% of the infants, slightly higher than that in our study (i.e., 3.6%) [16].

In a similar meta-analysis from Iran, including 42 studies and 18,000 premature infants, the prevalence of ROP was reported to be 23.5% [17]. It found that ROP prevalence was lowest in the west Iranian regions and most prevalent in the central region, showing the regional dependence of severity of ROP [17].

A review study from Latin America compiled the prevalence of ROP from the 33 studies published over a decade (between 2000 and 2010) in 10 countries and found it to range from 6.6% to 82%; the treatment requiring ROP ranged from 1.2% to 23.8% [18]. Its inclusion criteria differed in various regions of Latin American countries, ranging between birth weight thresholds of ≤1,500-2,000 grams and GA thresholds of ≤32-37 weeks. They also indicated that highly restrictive inclusion criteria should not be considered due to different social characteristics and variable neonatal care procedures [18].

LBW was observed in 98.9% of ROP babies in our study as compared to 91.5% in no ROP group with p value of 0.026. Prematurity was observed in 98.9% of ROP group and 82.3% of no ROP group with p value of 0.037. Both of these values are highly significant and correlate with almost all other studies.

As per an Indian study, a significant 10% of the patients who were relatively more mature also developed severe ROP, and 6.5% of the severe ROP cases had >2 kg birth weight [6]. This highlights that screening should be carried out even in mature infants or appropriate birth weight infants associated with other risk factors predisposing them to ROP. Sahu et al. also supported this notion in their study [19].

Apart from GA and birth weight, there is no clear consensus over the significant risk factors for ROP, likely due to regional variations in NICU infrastructure, the standard of neonatal care, and differing levels of exposure to potential contributing factors.

One study showed that oxygen administration is associated with the development of any ROP by three folds, while it increases the risk for severe ROP by seven folds [5]. Oxygen therapy, mechanical ventilation, and apnea were found significant in another study similar to ours [7]. In contrast, it also found septicemia to be independent risk factor for ROP [7]. Additionally, Meena et al. found significant correlation of oxygen administration with ROP and suggested its careful titration to avoid the related complications [4]. Furthermore, a Turkish study found median duration of oxygen therapy, sepsis, bronchopulmonary dysplasia (BPD) and IVH to be significantly associated with ROP [15].

Oxygen supplementation was not found a significant risk factor in a study from Kerala [9], likely due to regulated use of oxygen in that region and good NICU practices. However, another study from Kerala found oxygen supplementation to be a significant risk factor for ROP [10].

In our study, we observed that even infants with relatively lower birth weight and very low GA in the inborn group either did not develop ROP or developed only type 2 disease, which could be managed through close observation. In contrast, outborn infants with higher birth weight and GA often presented with more severe forms of ROP, such as AROP and hybrid ROP. These findings highlight the critical role of high-quality NICU care. As the survival rates of premature infants improve, so does the incidence of complications associated with prematurity, including severe ROP. One study has shown "outborn babies" being a significant risk factor for threshold and worse ROP [8], similar to our study.

RDS was found significantly associated with ROP in our study as well as in Meena et al.'s [4]. However, necrotizing enterocolitis (NEC), IVH were also found significant in their study, which were not found significant in our study. Similar correlation was observed in the study by Dwivedi et al. with RDS and low GA for AROP [6]. They observed late presentation for screening was significantly associated with Stage 4 and 5 disease development. However, they did not find any risk factor other than GA, LBW, and late presentation significant because they compared severe ROP with mild ROP [6], unlike in our study where we compared ROP with no ROP.

Anemia is another risk factor found significant in various studies [9,10] but not in ours. The number of blood transfusions was also found significant in the study by Goyal et al. [9]

In our study, phototherapy emerged as a statistically significant risk factor for the development of ROP (12 out of 15 infants who underwent phototherapy developed ROP) (p=0.001), a finding that has not been widely reported in previous literature.

NICU stay for more than two weeks was also found significant in our study with p=0.009. This can be understood by more unstable clinical course of children requiring longer hospitalization and being prone for developing ROP.

Multiple gestation was not found significant in our study or Rao et al.'s [11]. However, unlike our study, the latter found IVH as a significant risk factor for severe ROP [11].

In a Portuguese study, along with lower GA, hyperglycemia was also found a significant risk factor for severe ROP [14]. In a subgroup analysis of infants with GA 28 weeks or older, BPD, late onset sepsis, and hyperglycemia were linked to severe ROP [14].

Extremely LBW, pulmonary disease, IVH, and low GA were found to be significant risk factors for ROP in a Brazilian study [16] and an Iranian study [17]. They suggested that pulmonary diseases are markers for oxygen supplementation and fluctuations in oxygen concentration are associated with the pathogenesis of ROP development.

Vinekar et al. have shown that the sensitivity of American and British screening guidelines for picking threshold or worse ROP was 82.4% and 77.4%, respectively [8]. This highlights that in the developing countries like ours, regional guidelines should be followed to avoid missing the disease in larger and heavier infants as compared to their counterparts in developed countries. They also found septicemia as significant risk factor similar to the study by Gyoal et al. and Sahu et al. [10,19].

There is a paucity of published studies evaluating awareness of ROP among parents and caregivers in India. A study was conducted in Kerala by Bappal et al. on parents of infants undergoing ROP screening in rural areas [20]. After counseling them, a questionnaire was filled and it was found that 83.3% parents were aware that ROP is a blinding disease, as opposed to our study, where it was only 11.9%, which is likely because in our study counseling was done after filling the questionnaire. They also reported that 70% of the parents did not come for timely follow up as advised even after proper counseling.

Another similar study conducted in Turkey on the level of ROP awareness of parents, reported that 72% parents knew for which examination they had come but only 25% were aware of the blinding nature of ROP as opposed to our study where only 11.9% the parents knew about ROP and that it can cause blindness. Furthermore, in this study, only 31.5% parents had received information about ROP, most commonly from doctors and hospital staff during a prior NICU stay, which aligns with the findings of our study. It was emphasized in this study that as the education level of parents increased, the level of awareness about ROP also increased.

There are some studies on the awareness among pediatricians from Coimbatore, a two-tier city in south India indicating poor awareness. Only 65.1% (54) pediatricians were aware of ROP, and just 39.8% considered it preventable, while 28.9% believed it was not preventable. Notably, 41% did not know which part of the eye is affected, and 45.8% were unaware of the appropriate time to begin screening. Only 51.8% were confident that ROP is treatable. This emphasizes the need for increasing the awareness about ROP even in healthcare professionals for better screening and avoiding this potential blinding disease.

Limitations

This study has several limitations. First, being a cross-sectional study, it does not allow assessment of disease progression or regression, nor evaluation of dynamic factors such as the effects of rate of postnatal weight gain. Second, maternal and antenatal risk factors, as well as detailed oxygen exposure parameters (e.g., FiO₂, mode of respiratory support, and oxygen saturation), were not included, which may have influenced the outcomes. Third, due to regional variations in neonatal care, the findings may not be generalizable to other parts of the country. As this was a single-center tertiary care study including both inborn and referred outborn infants, referral bias cannot be excluded. It is possible that some eligible infants with fewer risk factors or relatively higher GA and birth weight were not referred for screening from peripheral healthcare facilities, which may have influenced the observed prevalence and severity of ROP. Despite these limitations, the study offers valuable insights into the spectrum of ROP in central India, and highlights the need to enhance awareness and screening practices.

## Conclusions

Due to the variability in neonatal care practices across regions, particularly in developing countries like India, western ROP screening guidelines cannot be directly applied. More mature infants, especially those with an unstable clinical course, remain at risk for ROP and should be considered for screening at the discretion of pediatricians. A collaborative approach involving pediatricians and ophthalmologists is essential for timely diagnosis and management. Strict monitoring and regulated use of supplemental oxygen are also crucial in preventing ROP, highlighting the need for standardized neonatal care protocols.

Improving outcomes in ROP requires investment in training healthcare providers, establishing screening capabilities in peripheral centers, and creating efficient referral systems to ensure early detection and treatment, even in resource-limited settings. Furthermore, targeted awareness programs for both parents and healthcare workers are essential to promote timely screening, consistent follow-up, and understanding of available treatment options. Such multifaceted efforts are key to reducing the burden of ROP-related blindness and ensuring equitable, sustainable care for at-risk infants.

## References

[1] Sai Kiranmayee P, Kalluri V (2019). India to gear up to the challenge of "third epidemic" of retinopathy of prematurity in the world. Indian J Ophthalmol.

[2] Sabri K, Ells AL, Lee EY, Dutta S, Vinekar A (2022). Retinopathy of prematurity: a global perspective and recent developments. Pediatrics.

[3] Chiang MF, Quinn GE, Fielder AR (2021). International classification of retinopathy of prematurity, third edition. Ophthalmology.

[4] Meena S, Bhatnagar K, Sheemar A, Gupta N, Tandon M, Agrawal N (2022). Unmet need for ROP screening in peripheral rural areas. Clin Ophthalmol.

[5] Vasavada D, Sengupta S, Prajapati VK, Patel S (2017). Incidence and risk factors of retinopathy of prematurity in western India - report from a regional institute of ophthalmology. Nepal J Ophthalmol.

[6] Dwivedi A, Dwivedi D, Lakhtakia S, Chalisgaonkar C, Jain S (2019). Prevalence, risk factors and pattern of severe retinopathy of prematurity in eastern Madhya Pradesh. Indian J Ophthalmol.

[7] Verma A, Nema N, Patel S, Ishrat S, Sidharth M (2016). Retinopathy of prematurity: incidence and risk factors. Int J Ophthalmol Eye Sci.

[8] Vinekar A, Dogra MR, Sangtam T, Narang A, Gupta A (2007). Retinopathy of prematurity in Asian Indian babies weighing greater than 1250 grams at birth: ten year data from a tertiary care center in a developing country. Indian J Ophthalmol.

[9] Lekha T, Balakrishnan D, Giridhar A, Alex D, Goyal A (2023). Retinopathy of prematurity in extreme preterm and extreme low-birth-weight infants: incidence, course, and risk factors. Middle East Afr J Ophthalmol.

[10] Goyal A, Giridhar A, Gopalakrishnan M, Thachil T (2019). Neonatal intensive care unit-based screening program for retinopathy of prematurity and its treatment in an Indian population. Indian J Ophthalmol.

[11] Rao KA, Purkayastha J, Hazarika M, Chaitra R, Adith KM (2013). Analysis of prenatal and postnatal risk factors of retinopathy of prematurity in a tertiary care hospital in South India. Indian J Ophthalmol.

[12] Nair A, El Ballushi R, Anklesaria BZ, Kamali M, Talat M, Watts T (2022). A review on the incidence and related risk factors of retinopathy of prematurity across various countries. Cureus.

[13] García H, Villasis-Keever MA, Zavala-Vargas G, Bravo-Ortiz JC, Pérez-Méndez A, Escamilla-Núñez A (2024). Global prevalence and severity of retinopathy of prematurity over the last four decades (1985-2021): a systematic review and meta-analysis. Arch Med Res.

[14] Almeida AC, Brízido M, Teixeira S, Coelho C, Borrego LM, Correia M (2022). Incidence and risk factors for retinopathy of prematurity in a Portuguese cohort. J Pediatr Ophthalmol Strabismus.

[15] Yucel OE, Eraydin B, Niyaz L, Terzi O (2022). Incidence and risk factors for retinopathy of prematurity in premature, extremely low birth weight and extremely low gestational age infants. BMC Ophthalmol.

[16] Freitas AM, Mörschbächer R, Thorell MR, Rhoden EL (2018). Incidence and risk factors for retinopathy of prematurity: a retrospective cohort study. Int J Retina Vitreous.

[17] Azami M, Jaafari Z, Rahmati S, Farahani AD, Badfar G (2018). Prevalence and risk factors of retinopathy of prematurity in Iran: a systematic review and meta-analysis. BMC Ophthalmol.

[18] Carrion JZ, Fortes Filho JB, Tartarella MB, Zin A, Jornada ID Jr (2011). Prevalence of retinopathy of prematurity in Latin America. Clin Ophthalmol.

[19] Sahu S, Behera S, Bishi G, Choudhury L, Mishra S (2024). Clinicoepidemiological profile of retinopathy of prematurity. Cureus.

[20] Bappal A, Jain R, Shambhu R, Peralaya K, Hegde V, Singh CB (2023). Awareness in parents of preterm babies screened and counselled for retinopathy of prematurity - a study from rural India. Kerala J Ophthalmol.

